# Sport sponsorship and sponsorship-linked investment under Saudi Vision 2030: a PRISMA-ScR review of direct and contextual evidence on brand equity, legitimacy, and value co-creation

**DOI:** 10.3389/fspor.2026.1920675

**Published:** 2026-09-24

**Authors:** Abdelrehim Awad, Ahmed Moustafa Aldabousi, Hebatalla E. Elbialy, Alaa A. Elnazer

**Affiliations:** 1 Department of Business Administration, College of Business, University of Bisha, Bisha, Saudi Arabia; 2 Umm Al Quwain University, Umm Al Quwain, United Arab Emirates; 3 Department of Management, Faculty of Business Administration, Horus University, New Damietta, Egypt; 4 Department of Finance, New Mansoura University, New Mansoura, Egypt; 5 Department of Marketing, College of Business, Imam Mohammad Ibn Saud Islamic University (IMSIU), Riyadh, Saudi Arabia

**Keywords:** brand equity, governance, legitimacy, PRISMA-ScR, Saudi Vision 2030, sport sponsorship, sportswashing, value co-creation

## Abstract

**Introduction:**

Saudi Arabia's Vision 2030 links sport sponsorship with commercial branding, sport-sector investment, tourism, public policy, and contested legitimacy narratives. This systematic scoping review maps direct evidence on sponsorship outcomes and distinguishes it from broader contextual scholarship.

**Methods:**

Following PRISMA-ScR and JBI guidance, an updated OpenAlex search was conducted on 22 August 2026 using 11 title-and-abstract query blocks, supplemented by backward/forward citation checking and targeted searches of official sources. Of 303 records identified (289 database hits and 14 supplementary records), 89 duplicates/duplicate versions were removed, leaving 214 records for screening. Thirty-seven full texts were assessed and 27 reports were included: six direct sponsorship studies and 21 contextual studies or reports. General theory sources were used only for interpretation.

**Results:**

Direct evidence, concentrated in football and dominated by cross-sectional, purposive, or qualitative designs, links sponsorship awareness and identification with sponsor image, attitude, purchase intention, and, more recently, stakeholder resistance to sponsor legitimacy. Contextual evidence maps modernization, soft-power, sportswashing, tourism, participation, and governance mechanisms but does not identify sponsorship effects. Study-level appraisal indicates moderate-to-high concern across most direct studies because of non-probability sampling, self-report measurement, narrow settings, or interpretive dependence.

**Discussion:**

The synthesis supports an evidence-calibrated framework in which audience processing explains near-term brand outcomes, while governance credibility and stakeholder participation are proposed boundary conditions for legitimacy and public value. These latter pathways, and the proposed measurement dashboard, remain author-derived propositions requiring direct empirical validation.

## 1 Introduction

Sport is one of the most visible components of the transformation of Saudi Arabia as part of its Vision 2030. Professional leagues, sports events around the world, club investment, esports, and tourism programs and community programs offer commercial opportunities for the sponsors, national-development opportunities, and a reputation-building opportunity. These activities are interdependent and not mutually exclusive. A sponsorship study can be conducted on a specific sponsor–property relationship, exposure, activation, or response of the audience; while a sport-investment, tourism, participation, or political-economy study can be based on the context in which sponsorship is understood.

If those units are conflated an attribution problem arises. Any positive effects that are claimed for the tourism and participation or for the league visibility or national reputation must be separated from the effects of the infrastructure, ownership, hosting of the event, media coverage and the wider policy reform, without which, there would be no positive effects. However, if the review were confined to six studies on direct sponsorship, certain institutional factors that might explain variations in the outcomes of similar sponsorship inputs for domestic and international actors would be omitted. The review therefore follows a very explicit two-tiered structure: direct evidence offers support for claims regarding the sponsorship relationships, while contextual evidence offers support for mechanisms, boundary conditions and research propositions but not for estimates of the effectiveness of sponsorship.

The review poses: RQ1: What is the amount, range and methodological profile of direct and contextual evidence relevant to the Saudi sport sponsorship in the context of the vision 2030? Which sponsorship exposure-audience processing-brand outcomes connections are directly supported by evidence (RQ2)? How do sources in the context define legitimacy, soft power, sportswashing, tourism, participation and governance, and how do they overlap or diverge? Which of the RQ4 relationships (in an integrated framework of brand equity and legitimacy and co-creation) are empirically supported and which are propositions that need to be researched?

The contribution is intentionally capped. Firstly, the review introduces a unit of analysis (only uses individually identifiable reports and separates direct and contextual evidence). Secondly, it evaluates each of the sources it provides with a suitable assessment tool for the design. Third, it develops a modal model of the relationship between sponsorship inputs and brand outcomes through audience processing, and the inputs of credibility of governance and participation of stakeholders and the outputs of legitimacy and co-created public value. This integration illuminates the context in which marketing salience can generate only short-term support that simply can't be achieved by the combination of brand-equity and legitimacy, and service-dominant-logic approaches.

## 2 Methods

### 2.1 Design and reporting

The study is a systematic scoping review (1, 2) that was reported following PRISMA-ScR (3) and informed by JBI guidance for scoping reviews (4). Pooled effects or certainty estimates are not made during the review, except in the items numbered (5) for which PRISMA 2020 items have been used in order to increase transparency. The narrative synthesis process was systematic, beginning with the grouping stage, followed by comparison within the group, convergence and divergence processes and ending with the interpretation of the strength of the story (6).

### 2.2 The scope of boundaries and evidence tiers

A direct sponsorship report needed to be one of the following: Saudi sport sponsor, sponsored property, sponsor activation, sponsorship exposure, or a sponsorship-specific audience outcome. A contextual report was required to explore Saudi sport investment, governance, tourism, participation, legitimacy, political economy etc, and to offer an explicit mechanism or boundary condition that could be relevant to the interpretation of sponsorship. No attempt was made to infer sponsorship effects from contextual reports. Interpretation was informed by general sponsorship theory, nation-brand theory and non-Saudi value-creation reviews were not accounted for in the PRISMA denominator. The operational eligibility criteria and evidence-tier rules are summarized in Table 1.

**Table 1:** Operational eligibility criteria and evidence-tier rules.

Element	Inclusion rule	Boundary/exclusion rule
Population/context	Saudi sport audiences, sponsors, properties, organizations, policymakers, tourists, or communities during the Vision 2030 period.	Saudi focus must be explicit; regional work required identifiable Saudi analysis.
Concept/exposure	Direct tier: sport sponsorship, sponsor activation, sponsor-property fit, awareness, or sponsor response. Contextual tier: sport investment/governance mechanisms demonstrably relevant to sponsorship interpretation.	Generic sport, tourism, or national-branding material without a sponsorship-relevant mechanism was excluded.
Outcomes	Brand awareness/equity/image, sponsor attitude or behavior; legitimacy/soft power/sportswashing; stakeholder participation, tourism, or public value.	Contextual outcomes were coded as conditions or propositions, not sponsorship effects.
Evidence types	Peer-reviewed empirical, qualitative, econometric, conceptual/critical, and review articles; official or industry reports with identifiable authorship/issuer and public accessibility.	Theory books/articles remained background references. Unpublished theses, working papers, and personal communications were excluded.
Language/time	English; 1 January 2016 to 22 August 2026.	Records published after the executed search date were outside the update.

### 2.3 Protocol

There was no protocol found that could be registered. It cannot replace registration as an alternative to the past, it is a methodological limitation. The eligibility boundary, evidence tiers, charting fields and appraisal rules below should be pre-registered in case of any changes that may be made in the future.

### 2.4 A collection of information sources and search strategy

The search for updates was automatically repeated using the OpenAlex Works API on 22 August 2026. Only the title_and_abstract.search field was used for searching and the search range was 01 Jan 2016 to 22 Aug 2026 (English). The 11 query blocks were for the following issues: Saudi context, sponsorship, football sponsorship, brand equity, legitimacy, sportswashing, tourism, investment, participation, esports and women sport. Using the identifier and comparing by title/DOI, 88 duplicate publications and one duplicate publication version were removed from the database, leaving 200 publications. There were 14 further non-duplicate eligible reports that were identified through backward/forward citation checking of eligible reports, and/or systematic search of the Saudi Vision 2030, Sport for All Federation and PwC publication sites. The supplementary PRISMA screening audit is developed and includes 214 records screened and decisions. The reproducible search strategy is provided in Appendix A, and the PRISMA-ScR reporting crosswalk is provided in Appendix B.

### 2.5 Selecting, calibrating and charting data

Selection was done by deduplication, abstract/titles screening and full-text evaluation. The screening and extraction at the revision stage were done by one reviewer who also used the prespecified operational rules; all the corpus was then rerun in reverse-chronological order and any inconsistencies in the decisions were checked. No inter rater agreement statistic or adjudication procedure is claimed as this is a repeated single-reviewer, not duplicate screening, audit. There were 4 types of decisions: include-direct, include-contextual, exclude at title/abstract, and exclude at full text/background theory. The following elements were included on the charting form: Citation, Route, Evidence Tier, Design, Sample or Document Corpus, Instrument or Data Source, Analytical Method, Extractable Result, Uncertainty, Limitations, and Appraisal Judgment. The complete 215 record audit comprised of the one version duplicate dropped prior to screening, 214 screened records, and mutually exclusive reasons for all exclusions. 2.6. The synthetic process and weighting with evidence.

### 2.6 The synthetic process and weighting with evidence

Synthesis of the direct and contextual tiers before integration. The following were compared: exposure, mechanism, outcome, design, direction, and magnitude of reported relationships, all of which were directly involved in the direct reports. All four of the following items were coded for the contextual reports: legitimacy frame, governance condition, stakeholder group, and temporal horizon and attribution strength. A claim was categorized as supported if it was measured directly in a study that investigated the sponsorship relationship. Only claims made only in the context were labeled as proposed, illustrative, or contested. No voting and no decision based upon the number of sources.

### 2.7 Study-level methodological appraisal

All sources included were reviewed at report stage by the same person who did the extraction. For this study, empirical qualitative, quantitative and mixed methods reports were evaluated using the design specific factors of the Mixed Methods Appraisal Tool (MMAT) 2018 (7). The six domains of SANRA (8) were used for the evaluation of narrative or critical reviews. The official and grey literature from the industry was assessed by authority, accuracy, coverage, objectivity, date and significance (9). Each judgment was made by a second pass to ensure accuracy with the reported design and also the full wording of the item. This is not reproduced independently, and therefore is not claimed as an adjudicator or agreement statistic, and is part of the limitations of the review. The judgments were: Yes, No, Partial, and Cannot tell and a cross design numerical total was not made.

### 2.8 Ethics and use of the language/AI tools

This was not an ethics approval review because it was a review of published documents and publicly available documents and there were no human participants and/or identifiable private documents involved. Grammarly was utilized to assist with editing language and formatting the audit files and manuscript during revision. All decisions are retained in the included record-level audit. The authors are solely responsible for the accuracy, originality, citations, eligibility boundary, appraisal judgments, interpretations and conclusions.

## 3 Results

### 3.1 Study selection

The 11 OpenAlex query blocks returned 289 hits. Deduplication removed 88 cross-query duplicates and one duplicate publication version, leaving 200 database records. Citation checking and targeted official-site searching added 14 unique supplementary reports, so 214 records underwent title/abstract screening. Of these, 177 were excluded because they lacked an eligible sponsorship relationship or a sufficiently specific contextual mechanism. Thirty-seven full texts evaluated with 10 excluded: four full text reports that were not published or finalized, three reports that were outside of the context/sponsorship boundaries with the study, two reports that lacked methodological evidence specific to Saudi Arabia or retrieval, and one preprint after the final text was identified. The final corpus consisted of 27 reports of which six were direct sponsorship reports and 21 were contextual reports. The corrected study-selection accounting is summarized in Table 2. A full decision log is provided as an audit file to supplement. The study-selection process is summarized in Figure 1.

**Table 2:** Corrected study-selection accounting.

PRISMA-ScR stage	Count (n)
OpenAlex query hits	289
Supplementary records	14
Duplicate hits/versions removed	89
Records screened	214
Records excluded at title/abstract	177
Full texts assessed	37
Full texts excluded	10
Included direct reports	6
Included contextual reports	21
Total included reports	27

**Figure 1:**
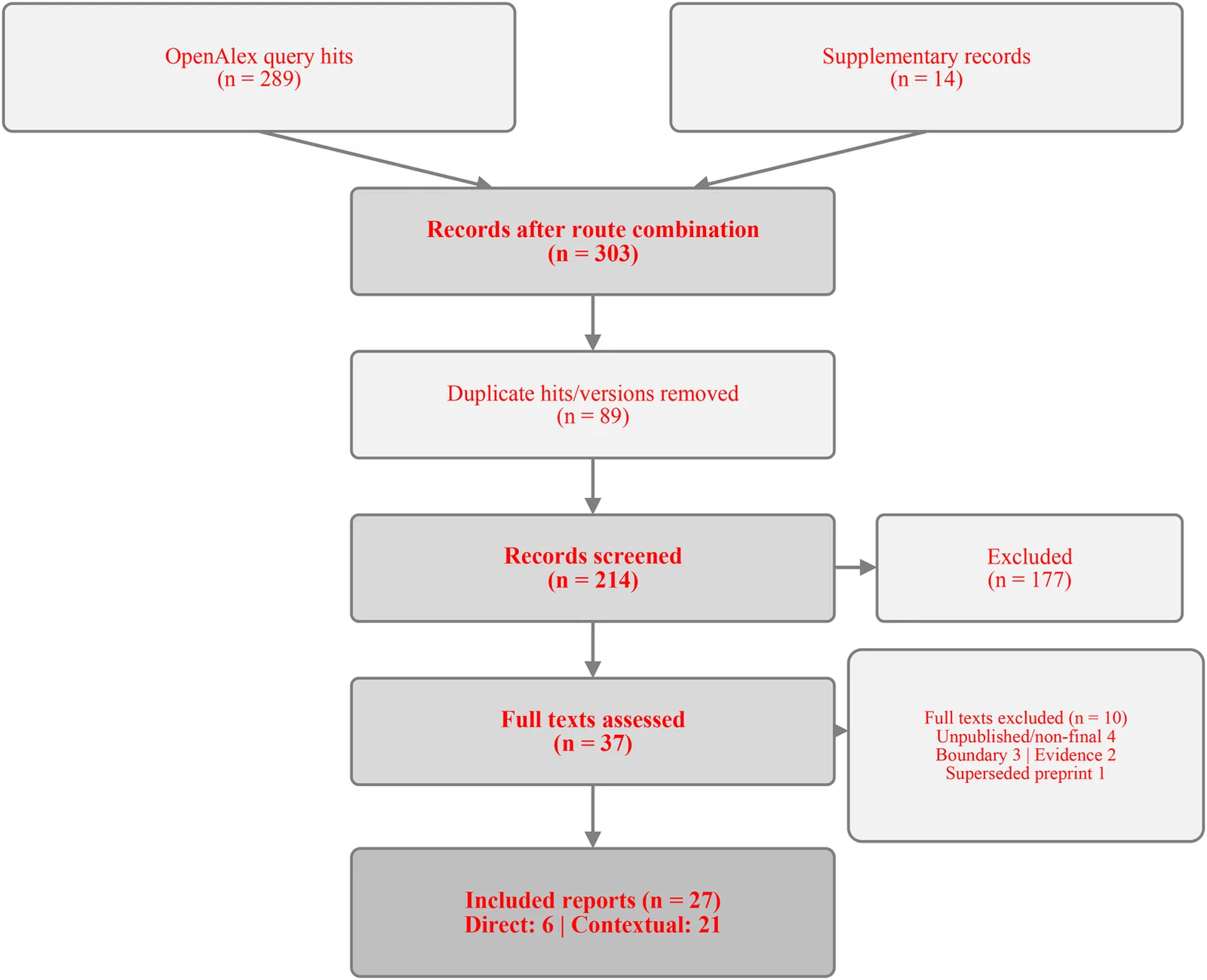
PRISMA-ScR flow diagram for the executed update search (22 August 2026).

### 3.2 Characteristics of direct sponsorship evidence

The direct tier contains six reports and seven empirical components because Alenazi (10) reports a survey and an experiment. Five studies examine audience or fan responses in Saudi football; Krieger and Schultz (11) analyze an athlete-led challenge to FIFA's Aramco sponsorship, extending the direct evidence from commercial response to sponsor legitimacy. The quantitative studies remain dominated by non-probability, convenience, and self-report designs, while the qualitative sponsorship-conflict study is interpretive rather than effect-estimating. The design, results, and limitations of the direct sponsorship studies are summarized in Table 3.

**Table 3:** Direct sponsorship studies: verifiable design, results, and limitations.

Study	Tier	Setting	Sample/design	Instrument/analysis	Extractable result	Individual limitations
Al-Nsour (12)	Direct	Riyadh sport audience	Cross-sectional purposive survey; *n* = 385	Questionnaire; correlation/regression	Brand enhancement → buying behavior: B = 0.758; r = 0.746; reported explained variance ≈ 54.9%	Non-probability, self-report, one city; causal language unsupported
Al-Nsour (13)	Direct	Riyadh journalists and fans	Purposive survey; journalists *n* = 40; fans *n* = 387	Questionnaire; group tests/regression	Positive association between sponsorship and brand-equity dimensions; age differences reported	Cross-sectional, geographically narrow, limited instrument reporting
Al-Nsour and Al-Sahli (14)	Direct	Roshn League audience	Purposive survey; *n* = 385	Structured 5-point questionnaire; model-fit/predictive analysis	Sponsorship awareness positively associated with sponsor brand image	Face-to-face convenience settings; same-source measures; no causal identification
Almaiman et al. (15)	Direct	Al Hilal–Tania sponsorship	Cross-sectional convenience survey; *n* = 190; 86% male	PLS-SEM; 10,000 bootstrap samples	Identification → awareness *β*=.251***; awareness → attitude β=.389***; awareness → purchase intention β=.188**; attitude → intention β=.552***	One club/low-tier sponsor; convenience sample; several single-item measures
Alenazi (10)	Direct	Saudi Pro League fans	Study 1 survey *n* = 563/600; Study 2 2 × 2 experiment *n* = 374	Survey models plus between-subjects sponsor-type × integration design	Processing fluency and fan benefits predict sponsor attitudes; naming rights and integration cues alter evaluations	Short-term attitudinal outcomes; allocation/manipulation and representativeness require cautious interpretation
Krieger and Schultz (11)	Direct	FIFA–Aramco sponsorship	Qualitative analysis of the open letter signed by more than 100 professional women footballers	Feminist standpoint analysis	Athlete marginalization and solidarity support an organized challenge to the sponsorship's institutional legitimacy	Single mobilization episode; interpretive analysis; no estimate of audience prevalence or sponsor effect

***p < .001; **p < .01. Coefficients are reported only where extractable from the source report. Associations are not interpreted as causal effects.

### 3.3 Direct sponsorship findings

#### 3.3.1 Convergence

Direct studies converge on a limited but coherent near-term sequence. There is a correlation between team identification and awareness of the sponsor (15), which in turn is correlated with the sponsor image, brand attitude, and purchase intention in various Saudi football samples (12–15). The most transparent model reports awareness → brand attitude (*β* = .389, *p* < .001), awareness → purchase intention (*β* = .188, *p* < .01), and brand attitude → purchase intention (*β* = .552, *p* < .001). The results are consistent with audience-processing and associative-transfer mechanisms, but they are mostly associational.

#### 3.3.2 Divergence and boundary conditions

The direct studies do not establish a uniform sponsorship effect. Both of these studies yielded only slight correlations between awareness and team performance, and sponsor type and integration cues modified the processing fluency and perceived fan benefits in Alenazi (10). Krieger and Schultz (11) find another route to be more qualitatively different, namely when athlete solidarity and perceived ethical conflict transform a high-visibility sponsorship into organized legitimacy resistance. So, response is conditioned by visibility, but it's not enough: Identification, integration, perceived fan value, sponsor conduct, and stakeholder values. There was no evidence in the literature that short-term commercial attitudes remain or extend to other sports, or that they lead to proven purchases, retention, or long-term brand equity; nor is there any estimate of how common legitimacy resistance is among stakeholder groups.

### 3.4 Contextual findings

Audiences and meanings of sport investment in Saudi Arabia are the main points agreed upon by contextual sources. Domestic accounts focus on modernization, participation, inclusion, tourism and opportunity while political-economy and critical accounts focus on state strategy, soft power, rights discourse and sportswashing. New evidence introduces four additional identifiable legitimacy mechanisms: media normalization, nostalgia, athlete resistance, and esports governance (11, 31, 32, 36). These are not exclusive effect findings but rather interpretations which are contingent upon the stakeholder(s) and the arena(s). No sport washing perceptions, cross-audience legitimacy or governance credibility are included as moderators in any of the quantitative direct sponsorship study analyses included in the reference list. Plausible downstream pathways are suggested by the tourism reports, participation reports and economic reports, but not necessarily by sponsorship. The constructs of competitive balance, disclosure, ownership arrangements and public-value reporting are thus considered as contextual conditions, which can potentially help or hinder legitimacy, but are not empirically proven mediators. This distinction is important because it allows for a distinction between what should be tested, and what is the effect of sponsorship. The characteristics, contributions, and attribution limits of the contextual evidence are summarized in Table 4. The report-level methodological appraisal of the included evidence is summarized in Table 5. The calibrated inferences across the principal claims are summarized in Table 6.

**Table 4:** Contextual evidence: data source, contribution, and attribution limit.

Source	Design/type	Context	Sample/data source	Permissible use in synthesis	Individual limitation
Ettinger (16)	Political-economy analysis	Sport diplomacy and investment	Published documents/secondary sources; corpus size NR	Contextual legitimacy/soft-power mechanisms	Interpretive framing and source-selection dependence
Svoboda et al. (17)	Qualitative exploratory study	Sport/physical activity reform	Qualitative corpus/participants as reported in source	Domestic meanings, modernization, legitimacy	Context-specific interpretation; transferability limits
Bataineh et al. (18)	Econometric secondary-data study	Saudi Pro League competitive balance	League performance data	Governance/competitive balance as reputation condition	No sponsorship exposure; structural breaks/confounding
Elshaer (19)	Quantitative survey	Football investment and quality of life	Fan sample; exact sampling details reported in source	Perceived quality-of-life association	Self-report; sponsorship not isolated
Kinawy (20)	Empirical tourism study	Football marketing/tourism	Tourism/football evidence reported in source	Destination visibility and tourism linkage	Attribution to sponsorship not identified
Alamri (21)	Case/survey	Eastern Region sport tourism	Regional respondents/data reported in source	Regional tourism-branding context	Single region; sponsorship exposure not isolated
Putra and Utomo (22)	Conceptual/document analysis	Saudi esports hub	Public documents; corpus NR	Nation-branding proposition	No sponsorship outcome or causal test
Joseph et al. (23)	Political-economy analysis	Gulf esports including Saudi context	Secondary/documentary evidence	Geopolitical and institutional positioning	Regional scope; Saudi-specific effects not separable
Imawan et al. (24)	Analytical/document study	Women's sport	Published/policy sources; corpus NR	Inclusion and empowerment narrative	Limited method transparency; no sponsorship test
Taylor et al. (25)	Critical narrative review	Arabian Peninsula sport	Regional literature corpus	Maps global/local legitimacy and sportswashing debate	Not systematic; heterogeneous regional evidence
Almdoudi and Alalmai (26)	Macro regression	PIF sport revenue/economic development	Secondary time-series/aggregate data	Macro association	Endogeneity, lag choice, sponsorship not separable
PwC (27)	Industry report	Middle East sport market	Proprietary/compiled market data	Descriptive market context	Commercial authorship; methods and uncertainty incompletely reported
Saudi Vision 2030 (28)	Official report	National sport evolution	Administrative/policy reporting	Official objectives and descriptive indicators	Institutional advocacy; not independent effectiveness evidence
Sport for All Federation (29)	Official strategy	Participation/community sport	Strategic and administrative information	Participation/inclusion policy context	Strategic document; no counterfactual or sponsorship attribution
Alsharif et al. (30)	Mixed-methods study	Women's sport under Vision 2030	Survey *n* = 138; five semi-structured interviews	Institutional and community legitimacy conditions	Participation rather than sponsorship outcome; localized sample
Rogstad (31)	Qualitative media analysis	Saudi state involvement in esports	53 Norwegian news articles, 2020–2024	Esportswashing normalization and governance mechanism	Single national media system; framing is not stakeholder prevalence
Farrell-Banks and Merrill (32)	Comparative media/discourse analysis	Newcastle United takeover	Twitter and British newspaper discourse during the first post-takeover year	Nostalgic legitimation/delegitimation mechanism	Platform and media-selection dependence; sponsorship effect not isolated
Alghamdi (33)	Critical book chapter	Saudi sport events	Published conceptual and documentary sources	Legitimate development versus sportswashing boundary	Non-systematic source selection; no measured sponsorship outcome
AlSheddi (34)	Qualitative interview study	Football and nation branding	12 expert interviews across six disciplinary fields	Modernization, soft-power, and reputation mechanisms	Expert perceptions; no public-audience or sponsorship-effect estimate
Awad (35)	Systematic narrative/conceptual synthesis	Saudi mega-event marketing	92 peer-reviewed and institutional sources	Credibility conditions connecting event experience, digital activation, and nation-brand image	Author overlap with the present review; conceptual pathways require primary testing
Kim and Sakib (36)	Qualitative plus quantitative media-framing analysis	Esports soft power across four countries	Newspaper content from China, Saudi Arabia, South Korea, and the United States	Cross-national media framing of diplomacy and sportswashing	Media frames are not direct measures of stakeholder legitimacy

NR, not reported or not applicable. Contextual reports inform mechanisms or boundary conditions only; they do not enter claims about sponsorship effects.

**Table 5:** Individual report-level methodological appraisal.

Source	Tool/design	Clear question	Sampling/source fit	Measurement/data credibility	Analysis traceable	Conclusions bounded	Overall concern
Al-Nsour (12)	MMAT-QN	Yes	No	Cannot tell	Yes	No	High
Al-Nsour (13)	MMAT-QN	Yes	No	Cannot tell	Yes	No	High
Al-Nsour and Al-Sahli (14)	MMAT-QN	Yes	No	Cannot tell	Yes	No	High
Almaiman et al. (15)	MMAT-QN	Yes	No	Partial	Yes	Partial	Moderate–high
Alenazi (10)	MMAT-QN/QE	Yes	Partial	Partial	Yes	Partial	Moderate
Ettinger (16)	SANRA	Yes	Partial	Partial	Yes	Partial	Moderate
Svoboda et al. (17)	MMAT-QL	Yes	Partial	Yes	Yes	Partial	Moderate
Bataineh et al. (18)	MMAT-QN	Yes	Yes	Partial	Yes	Partial	Moderate
Elshaer (19)	MMAT-QN	Yes	Partial	Cannot tell	Partial	No	High
Kinawy (20)	MMAT-QN	Yes	Partial	Cannot tell	Partial	No	High
Alamri (21)	MMAT-QN	Yes	Partial	Cannot tell	Partial	No	High
Putra and Utomo (22)	SANRA	Yes	Partial	Partial	Partial	No	Moderate–high
Joseph (23)	SANRA	Yes	Yes	Partial	Yes	Partial	Moderate
Imawan et al. (24)	SANRA	Yes	Partial	Cannot tell	Partial	No	High
Taylor et al. (25)	SANRA	Yes	Yes	Partial	Yes	Partial	Moderate
Almdoudi and Alalmai (26)	MMAT-QN	Yes	Partial	No	Partial	No	High
PwC (27)	AACODS	Yes	Partial	Partial	Partial	Yes	High for effectiveness
Saudi Vision 2030 (28)	AACODS	Yes	Partial	Partial	No	Yes	High for effectiveness
Sport for All Federation (29)	AACODS	Yes	Partial	Partial	No	Yes	High for effectiveness
Krieger and Schultz (11)	MMAT-QL	Yes	Partial	Yes	Yes	Partial	Moderate
Alsharif et al. (30)	MMAT-MM	Yes	Partial	Partial	Yes	Partial	Moderate
Rogstad (31)	MMAT-QL	Yes	Partial	Yes	Yes	Partial	Moderate
Farrell-Banks and Merrill (32)	MMAT-QL	Yes	Partial	Partial	Yes	Partial	Moderate
Alghamdi (33)	SANRA	Yes	Partial	Partial	Partial	Partial	Moderate–high
AlSheddi (34)	MMAT-QL	Yes	Partial	Yes	Yes	Partial	Moderate
Awad (35)	SANRA	Yes	Yes	Partial	Yes	Partial	Moderate
Kim and Sakib (36)	MMAT-MM	Yes	Partial	Partial	Yes	Partial	Moderate

MMAT-QN = quantitative nonrandomized; MMAT-QE = quantitative experimental; MMAT-QL = qualitative. ‘Overall concern’ is a narrative judgment, not an arithmetic score. The full item wording follows Hong et al. (7), Baethge et al. (8), and Tyndall (9).

**Table 6:** Convergence, divergence, and calibrated inference.

Claim	Evidence pattern	Counter-evidence/uncertainty	Permissible inference
Awareness → sponsor evaluations	Converges across four direct reports	Self-report and non-probability samples	Supported association; causal effect not established
Identification/fit/integration	Supported as audience-processing conditions	Different measures and properties	Probable moderators/antecedents; replication needed
Legitimacy/sportswashing	One qualitative direct study documents athlete resistance; contextual sources identify competing frames	No prevalence estimate or quantitative moderator test	Demonstrated legitimacy conflict in one case; general effect remains untested
Value co-creation/public value	Frequently proposed in contextual evidence	Behavioral and longitudinal measures absent	Provisional pathway requiring direct tests
Tourism/economic development	Macro and policy sources report positive development narratives	Sponsorship not separated from investment and policy	No attribution to sponsorship

### 3.5 Evidence-calibrated integrated framework

Figure 2 distinguishes tested and proposed links. Solid paths summarize direct evidence: sponsorship inputs are processed through awareness, identification, fluency, fit, and perceived benefits, producing near-term sponsor-image, attitude, and intention outcomes. Dashed paths are propositions derived from contextual evidence: governance credibility and stakeholder participation may shape legitimacy and public value. The model's theoretical novelty is conditional integration: legitimacy is not another parallel outcome but a boundary condition that may strengthen or weaken the conversion of sponsorship salience into durable stakeholder support; co-creation is the participatory process through which sponsor and public outcomes may become mutually reinforcing.

**Figure 2:**
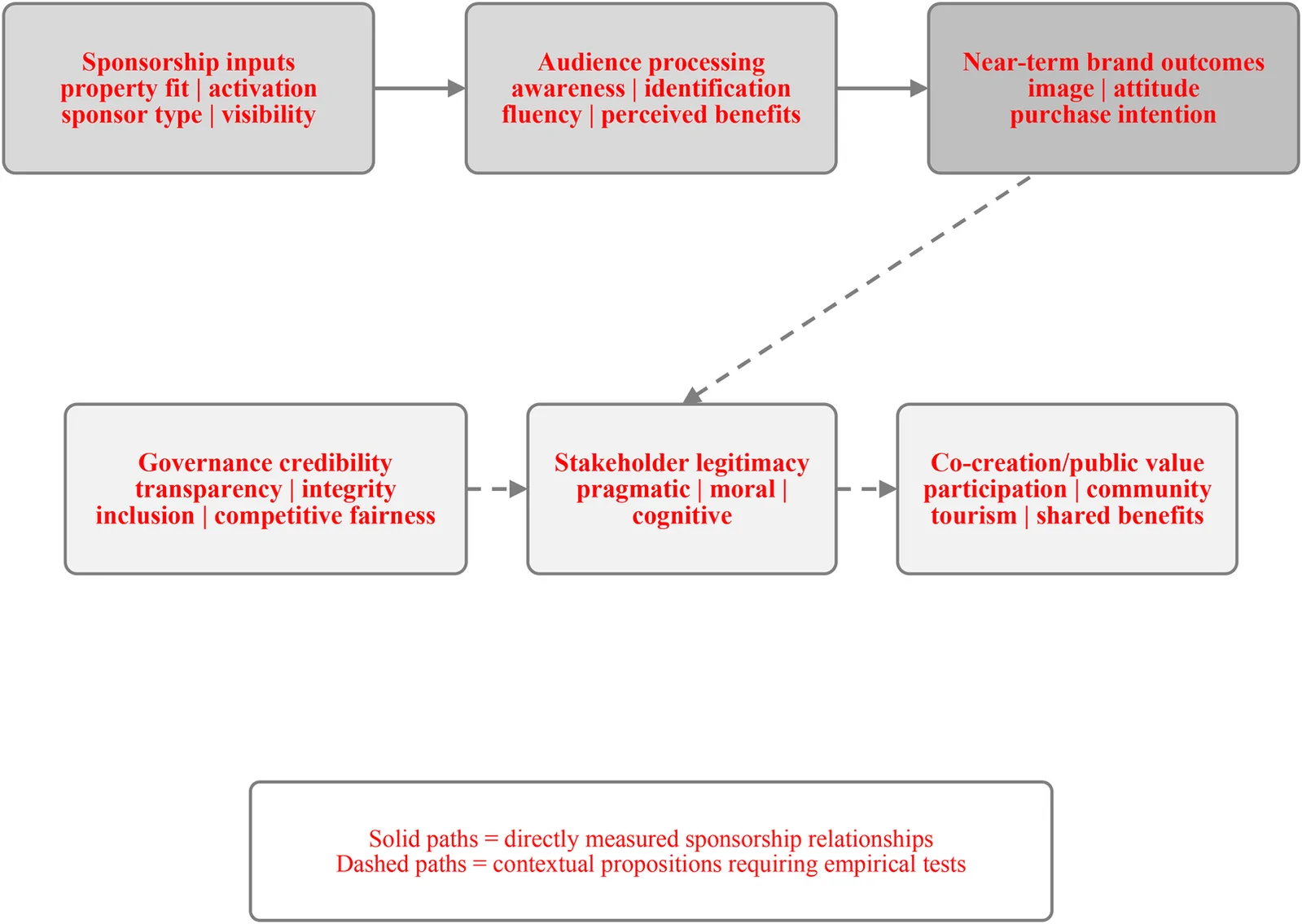
Evidence-calibrated mechanism framework. The figure is author-generated from the tiered synthesis and is a testable proposition, not a validated causal model.

### 3.6 Provisional measurement architecture

The dashboard is recast as a provisional measurement architecture. It is not an instrument validated by the included literature. Indicators are mapped to constructs and evidential status so that future evaluations can distinguish direct sponsor outcomes from contextual public-value claims. The provisional measurement architecture and evidential status are summarized in Table 7.

**Table 7:** Provisional measurement architecture and evidential status.

Construct	Candidate indicators	Preferred data	Status in current evidence
Audience processing	Unaided/aided awareness; identification; fit; fluency; perceived fan benefit	Survey plus recognition task/experimental manipulation	Direct evidence; standardization needed
Brand outcomes	Sponsor image; attitude; purchase intention; verified purchase/renewal	Brand tracking, CRM, transaction or panel data	Attitude/intention supported; behavior largely absent
Legitimacy	Trust, transparency, fairness, inclusion, integrity, sportswashing perception	Multi-stakeholder surveys, media coding, interviews	Contextual proposition; no direct Saudi sponsorship test
Co-creation	Activation participation, UGC, repeat attendance, volunteering, community enrollment	Platform logs, event/program records, longitudinal surveys	Contextual proposition; behavioral evidence absent
Public value	Tourism conversion, local spending, participation, distributional benefit	Administrative data with comparison groups and lag models	Do not attribute without counterfactual design

## 4 Discussion

### 4.1 What the evidence does support

The defensible conclusion is smaller than a broad assertion that Vision 2030 sport sponsorship fosters brand equity, legitimacy and public value. The relationships between these factors are supported by direct quantitative evidence related to the Saudi football context, and the results indicate that the type of sponsors, integration, processing fluency, and the perceived benefits of the fans are key factors. Direct evidence is another form of qualitative evidence that shows that when there is an ethical conflict, the athlete resistance to the legitimization of the sponsor may be activated. The evidence is overwhelmingly often reported from non-probability, cross-sectional, self-reported or single case interpretive designs, and hence, cannot be used to directly identify causal effects or generalise to other sports, sponsors, stakeholders or regions.

### 4.2 A theoretical contribution of conditional integration is desired, not additive

The brand-equity theory can explain how the same brand association can generate associative salience and evaluative transfer (37–45); the legitimacy theory can explain why associative salience and evaluative transfer of the same brand association may be accepted by some, but not others (46–51); and the value co-creation theory can explain how a value co-creation can transform a one-way communication investment into a two-way experience and benefit (52–59). The integrated model takes the claim of audience processing a step further: Areas of audience processing yield short-term marketing results; however, the ability of governance to be credible is a prerequisite to areas of audience processing yielding long-term marketing results that are sustained by the audience and are participative activation, or are created by the audience themselves. This conditional sequence is more clear than if four theories were lined up next to each other. The model also allows for predictions that can be tested. The link between positive attitude of the sponsors and multi-stakeholder legitimacy should be strengthened by transparency in governance, and the sportswashing perceptions should weaken it. Participation in the activation should be “trans-mediated” with the effect on perceived fan benefit on co-created value. The propositions are not reported as findings since there is no direct study included in the work that tests these propositions.

### 4.3 Implications for practice and governance

Sponsors have the power to make a positive impact based on direct evidence by enhancing recognizability, property fit, integration and measurable fan value. They should not make any assumptions about tourism and inclusion effects or national-reputation effects based on awareness data. Brand and governance measurements and independent stakeholders’ measurements should be considered together when assessing sport properties. The rules of attribution logic should be made a requirement for policy makers: Public value claims must be based on a baseline, provided a comparison group if applicable, set a reasonable time horizon and differentiate between the sponsoring of the event and the growth of the capital investment and the impact of hosting the event. The evaluation minimum portfolio is then layered out: audience-processing measures, sponsor outcomes, stakeholder legitimacy and participatory behaviour, and public-value indicators. The final three layers do not represent a measure-based dashboard, but a proposed measure program.

### 4.4 Limitations of the review

This review was not registered in advance. The reproducible update was done based on one broad open bibliographic index, some targeted and official site searches for citations, and may have overlooked some relevant records that were poorly indexed. A two-pass repeated single-reviewer audit was used instead of duplicate review to achieve the screening, extraction and appraisal. Restriction to English may be a reason to discontinue Arabic scholarship. Football-dominant and the direct corpus is small and heterogeneous. One contextual synthesis was written by the present lead author and went through the same appraisal rules, and was not used as effect evidence. Study-level appraisal continues to be design sensitive and is not designating equivalency for unlike evidence types. There were no appropriate metanalyses and a causal or certainty of effect statement is not provided.

### 4.5 Research agenda

Priority designs include those based on preregistered longitudinal panels around sponsorship announcements and activation phases, randomized or quasi-experimental experimental tests of the type of sponsorship, sponsorship fit, integration and disclosure, multi-stakeholder comparisons of domestic and international legitimacy, difference-in-differences designs, and synthetic-control designs for tourism and public-value outcomes. Research needs to be expanded to women's football, sports for the disabled, community programmes, regional clubs and esports.

## 5 Conclusion

Saudi sport sponsorship is an important field of research, yet it is still in the early stages of development, from a methodical point of view. There are six direct reports to the near term audience processing pathway of identification and awareness to the image/attitude of the sponsor to intention to athlete resistance, and no general impacts on tourism and public value. Twenty-one contextual reports are provided to explain why governance, stakeholder interpretation, participation, political economy and media framing may be important, but have yet to have been tested in designs for sponsorships. The review covers the three types of information (direct evidence, contextual and background theory) that are then clearly differentiated, and then unsupported links (propositions) are clearly identified. The next step is to make more compelling claims, derived from the measurement of multi-stakeholders, rather than more claims, derived from widely variable evidence.

## Data Availability

The original contributions presented in the study are included in the article/Supplementary Material, further inquiries can be directed to the corresponding author.

## Appendix

### Appendix A Reproducible search strategy

**Table A1:** Platform-specific search reporting.

Source	Fields	Search syntax	Limits/coverage	Date/yield
OpenAlex Q1	title_and_abstract.search	"Saudi Arabia” sport sponsorship	2016-01-01 to 2026-08-22; English	22 Aug 2026; *n* = 12
OpenAlex Q2	title_and_abstract.search	"Vision 2030” sport sponsorship	Same limits	22 Aug 2026; *n* = 3
OpenAlex Q3	title_and_abstract.search	"Saudi Arabia” football sponsorship	Same limits	22 Aug 2026; *n* = 11
OpenAlex Q4	title_and_abstract.search	"Saudi Arabia” sport “brand equity"	Same limits	22 Aug 2026; *n* = 1
OpenAlex Q5	title_and_abstract.search	"Saudi Arabia” sport legitimacy	Same limits	22 Aug 2026; *n* = 17
OpenAlex Q6	title_and_abstract.search	"Saudi Arabia” sportswashing	Same limits	22 Aug 2026; *n* = 46
OpenAlex Q7	title_and_abstract.search	"Saudi Arabia” sport tourism	Same limits	22 Aug 2026; *n* = 22
OpenAlex Q8	title_and_abstract.search	"Saudi Arabia” sport investment	Same limits	22 Aug 2026; *n* = 45
OpenAlex Q9	title_and_abstract.search	"Saudi Arabia” sport participation	Same limits	22 Aug 2026; *n* = 57
OpenAlex Q10	title_and_abstract.search	"Saudi Arabia” esports	Same limits	22 Aug 2026; *n* = 39
OpenAlex Q11	title_and_abstract.search	"Saudi Arabia” women sport	Same limits	22 Aug 2026; *n* = 36
Supplementary routes	Backward/forward citations and targeted official sites	Reference/citing records of eligible studies; Saudi Vision 2030, Sport for All Federation, and PwC publication pages	Non-duplicate eligible records only	22 Aug 2026; *n* = 14

Across the 11 OpenAlex blocks, 289 query hits were retrieved. Cross-query deduplication removed 88 hits; title/DOI comparison removed one publication-version duplicate. The 14 supplementary records were unique, producing 214 screened records. The raw export, query summary, and record-level decision audit accompany the manuscript.

### Appendix B PRISMA-ScR reporting crosswalk

**Table B1:** PRISMA-ScR checklist crosswalk.

Item	Requirement	Location	Status
1	Title identifies scoping review	Title	Reported
2	Structured summary	Abstract	Reported
3	Rationale	Introduction	Reported
4	Objectives/questions	Introduction	Reported
5	Protocol/registration	2.3	No registration; disclosed
6	Eligibility criteria	2.2 and Table 1	Reported
7	Information sources	2.4 and Appendix A	Reported with exact date and yields
8	Search	Appendix A	Exact executed strings reported
9	Selection of sources	2.5	Single-reviewer two-pass audit disclosed
10	Data charting	2.5	Procedure and fields reported
11	Data items	2.5; Tables 3, 4	Reported
12	Critical appraisal	2.7; Table 5	Single-appraiser two-pass procedure disclosed
13	Synthesis methods	2.6	Reported
14	Selection results	3.1; Figure 1	Counts and mutually exclusive reasons reported
15	Characteristics of sources	Tables 3, 4	Reported
16	Critical appraisal within sources	Table 5	Reported
17	Results of individual sources	Tables 3, 4; 3.3–3.4	Reported
18	Synthesis results	3.3–3.6	Reported
19	Summary of evidence	4.1–4.3	Reported with tiering
20	Limitations	4.4	Reported
21	Conclusions	Conclusion	Reported
22	Funding	Funding	Reported
